# The UPDATE trial (*UVB* *P*hototherapy in *D*ermatology for *AT*opic *E*czema): study protocol for a randomized controlled trial of narrowband UVB with optimal topical therapy versus optimal topical therapy in patients with atopic eczema

**DOI:** 10.1186/s13063-024-08334-z

**Published:** 2024-07-16

**Authors:** Eva Knöps, Phyllis Spuls, Ruben Duijnhoven, Marcel Dijkgraaf, Marit van Barreveld, Bernd Arents, Annefloor van Enst, Floor Garritsen, Maruschka Merkus, Maritza Albertina Middelkamp-Hup, Annelie Musters, Angela Bosma, Ariënna Hyseni, Jitske Dijkstra, Dirk Jan Hijnen, Louise Gerbens

**Affiliations:** 1 Department of Dermatology, Amsterdam UMC, Location Academic Medical Center, Amsterdam Public Health, Infection and Immunity, University of Amsterdam, Amsterdam, The Netherlands; 2 Department of Obstetrics and Gynecology, Amsterdam UMC, Amsterdam Reproduction & Development Research Institute, University of Amsterdam, Amsterdam, the Netherlands; 3 Department of Epidemiology and Data Science, Amsterdam University Medical Centers, University of Amsterdam, Amsterdam, the Netherlands; 4 Dutch Association for People With Atopic Dermatitis, Nijkerk, the Netherlands; 5 Nederlandse Vereniging Voor Dermatologie en Venereologie, NVDV, Utrecht, the Netherlands; 6 Department of Dermatology, HagaZiekenhuis, The Hague, the Netherlands; 7 Department of Dermatology, Erasmus MC University Medical Center, Rotterdam, the Netherlands

**Keywords:** Atopic eczema, Atopic dermatitis, Phototherapy, NB-UVB, Topical therapy, Optimal topical therapy, Effectiveness, Cost-effectiveness, Safety, Randomized controlled trial

## Abstract

**Background:**

Narrowband ultraviolet B (NB-UVB) phototherapy is commonly prescribed for patients with moderate-to-severe atopic eczema (AE). The efficacy of NB-UVB, however, has not yet properly been established, as current evidence is of low certainty. Our aim is to assess the short-term and long-term (cost-)effectiveness and safety of NB-UVB in adult AE patients by performing a pragmatic, multicenter, prospective, randomized, open-label, blinded-endpoint (PROBE) trial. This protocol outlines its methodology.

**Methods:**

A pragmatic, multicenter, PROBE trial will be performed with 1:1 randomization of 316 adult patients with moderate-to-severe AE who have inadequate disease control with topical therapy and who are eligible for optimal topical therapy (OTT) or NB-UVB in combination with OTT as a next step. Participants in the interventional arm will receive a minimum of 3 months of OTT combined with 8 to 16 weeks of NB-UVB. The control group receives 3 months of OTT. Following the interventional phase, follow-up will continue for 9 months. Physician-reported and patient-reported outcomes (according to the Harmonising Outcome Measures for Eczema (HOME) Core Outcome Set) and adverse events are assessed at 4 weeks, 3, 6, 9, and 12 months.

**Discussion:**

The UPDATE trial aims to provide high-quality evidence regarding the (cost-)effectiveness and safety of NB-UVB phototherapy in moderate-to-severe AE patients. Challenges that are addressed in the protocol include the possible bias arising from applying open-label treatment and the necessity of introducing OTT into the study design to prevent a high dropout rate.

**Trial registration:**

ClinicalTrials.gov NCT05704205. Registered on December 8, 2022.

**Supplementary Information:**

The online version contains supplementary material available at 10.1186/s13063-024-08334-z.

## Introduction

### Background and rationale

Atopic eczema (AE) (syn. atopic dermatitis) is a dermatological condition characterized by a chronic fluctuating pruritic inflammation of the skin. It is a common disease that affects 2–10% of the adult population, thereby posing a high burden in terms of health care costs [1–3]. According to the World Health Organization (WHO) Global Burden of Disease project, AE is in the top 50 of the most prevalent diseases worldwide and is the leading skin condition regarding disease burden measured by disability-adjusted life years (DALYs) [4].

In clinical practice, AE can be treated using a stepped-care approach [5, 6]. Patients whose AE is insufficiently controlled by standard topical care are eligible for optimal topical therapy (OTT), phototherapy, or systemic immunomodulating therapy.

OTT entails a combination of a personalized topical regimen with detailed instructions (including the fingertip unit method) and lifestyle recommendations, including advice regarding washing/bathing, avoidance of triggers and contact allergens, and psychological support. In the 2022 European guideline (EuroGuiDerm) on AE recommendations and suggestions for the optimal topical care of AE patients based on expert consensus can be found [5, 6].

Phototherapy is a frequently initiated first-line therapy for moderate-to-severe AE; it is prescribed by approximately 85% of European dermatologists as highlighted by a survey performed in 2018. Types of phototherapy include, i.e., photochemotherapy (PUVA), ultraviolet A-1 (UVA1), broadband ultraviolet B (BB-UVB), and narrowband ultraviolet B phototherapy (NB-UVB). The 2018 survey clarified that NB-UVB is the most commonly applied type (80.9%) [7]. NB-UVB devices contain fluorescent lamps emitting UVB in the 311 nm to 313 nm range. Guidelines on the dosimetry of NB‐UVB have mainly been published for psoriasis [8–12], but these dosing protocols are often also used for AE, as dosing studies for AE are not available.

High-quality evidence for the efficacy, (cost-)effectiveness, and safety of NB-UVB, however, does not exist for AE. Remarkably, NB-UVB for AE has only been studied in few short-term (9–12 weeks) and low-to-moderate-quality randomized controlled trials (RCTs) [13]. Recently, a Cochrane systematic review assessed the currently available evidence on the different phototherapy options for AE [14]. It concluded that NB-UVB is safe and effective in AE patients and may improve physician-assessed AE signs and patient‐reported symptoms when compared to placebo. However, firm conclusions could not be drawn due to the available evidence being of very low to low certainty. It therefore remains unclear whether NB-UVB is (cost-)effective for the treatment of AE.

Recently, this lack of evidence has resulted in the imminent discontinuation of reimbursement of phototherapy for AE in the Netherlands by some health insurance companies [15]. Discontinuation of reimbursement of NB-UVB will lead to the loss of this therapeutic option. This may lead to a shift to new on-label and much more expensive systemic treatments (each with its own safety profile) that have been proven effective in RCTs. Besides, patients and dermatologists often hesitate to initiate systemic therapy (reasons include but are not limited to the costs of these treatments and the possibility of side effects) and hope to postpone this next step with phototherapy. In some patients, such as patients with a child wish, many topical and systemic therapies are not suitable. It is therefore important that this type of phototherapy is further investigated in a well-designed RCT to establish if NB-UVB should still have a place in the treatment algorithm.

### Objectives

The aim of the UPDATE trial is to investigate the short-term and long-term effectiveness, cost-effectiveness, and safety of NB-UVB + OTT versus OTT alone in the treatment of adult patients with moderate-to-severe AE who have inadequate disease control with topical therapy and who are eligible for OTT or NB-UVB + OTT as a next step. This will be investigated in a pragmatic, multicenter, prospective, randomized, open-label, blinded-endpoint (PROBE) trial with 1:1 allocation in a superiority framework [16].

## Methods and trial design

### Study setting

This study will be conducted in Dutch secondary and tertiary care centers. The listing of participating clinics and hospitals can be found on www.ClinicalTrials.gov, identifier NCT05704205.

### Eligibility criteria

Adult patients with moderate-to-severe AE who have inadequate disease control with their current topical treatment will be included in this study. A variety of topical treatments may have been tried including topical corticosteroids, calcineurin inhibitors, coal tar preparations, and emollients with more or less specific instructions due to the limited time in most secondary care settings. They are eligible for OTT or NB-UVB + OTT as a next step.

### Inclusion criteria

In order to be eligible to participate in this study, a subject must meet all of the following criteria:

- Adult (≥ 18 years of age) patient meeting the UK working party criteria for AE [17];
- AE insufficiently controlled by standard topical care and therefore eligible for OTT or NB-UVB;
- Validated Investigator Global Assessment for Atopic Dermatitis (vIGA-AD) of ≥ 2 (moderate disease);
- Eczema Area and Severity Index (EASI) of ≥ 7 (moderate disease); and
- Ability to provide written informed consent.

### Exclusion criteria

A potential subject who meets any of the following criteria will be excluded from participation in this study:

- Contra-indication for NB-UVB [18];
  - Genetic defects associated with photosensitivity or skin cancer;
  - Heavily photo-damaged skin;
  - History of melanoma or non-melanoma skin malignancies (in case of basal cell carcinoma patient is excluded if one has a history of > 2 basal cell carcinomas);
  - Use of systemic immunosuppressants/immunomodulators;
  - Use of medication associated with photosensitivity;
- Patient is already on systemic AE therapy;
- Patient is already on OTT for the past 2 months; and
- NB-UVB or any systemic therapy with influence on AD in the past 9 months.

A course of prednisolone of 15 days or more is regarded as systemic AE therapy.

### Informed consent

Subjects will be recruited from the patient population of each participating center. Possible eligible patients will be informed verbally about the study by a trained investigator. This may be in person or via telephone/video call. The patient will receive the participant information sheet and informed consent form (ICF) digitally or in print. Patients who agree to participate will be asked for written informed consent after reviewing the abovementioned information during a reflection period of at least one hour. In order to schedule the screening activities, it is allowed that the patient gives oral consent. The oral consent must be documented in the digital patient file. At the appointment of the screening activities the patient and the investigator will sign the informed consent form together. Allocated therapy cannot start before both parties have signed the ICF.

On the consent form, participants will be asked if they agree to the usage of their data should they choose to withdraw from the trial. Participants will also be asked for permission for the research team to share data to investigators, auditors, or regulatory authorities, where relevant. No ancillary studies are planned. This trial does not involve collecting biological specimens for storage.

### Interventions

We will investigate a course of NB-UVB + OTT (home or out-patient) of 12 weeks (range of at least 8 weeks and up to 16 weeks) compared to OTT alone. In the interventional arm, NB-UVB will be given 3 times per week with daily OTT. In the control group daily OTT will be applied.

#### Intervention description

##### NB-UVB

Both outpatient clinic and at-home NB-UVB will be assessed. The ultraviolet emission from phototherapy booths and panels is made possible by UVB lamps. NB‐UVB devices contain fluorescent lamps emitting UVB in the 311 nm to 313 nm range [19].

In the out-patient clinic administration of NB-UVB rays will be executed according to the manufacturer’s instructions by trained nurses. Patients undergoing at-home therapy will receive detailed instructions on the treatment procedure prior to starting NB-UVB. During the course participants will be remotely monitored via the site’s affiliated phototherapy panel supplier, following the local procedure. During administration, all patients will wear protective goggles and cover the genital area to shield the eyes and scrotum/vulva from ultraviolet rays. Administration will occur three times a week.

The patient’s initial dosing and dosage modification during phototherapy will be done according to a standardized study dose regimen (Additional file 2). During NB-UVB treatment, the researcher (or if the researcher is not a physician: the researcher together with a supervising physician) will monitor therapy response and tolerability and determine treatment duration (minimum of 8 weeks, maximum of 16 weeks).

After completing the NB-UVB course and applying 3 months of OTT (as listed below), patients are allowed to take the next step in the AE treatment algorithm if needed, being one of the currently available systemic immunomodulating therapies.

##### OTT

All participants will use daily OTT. This consists of detailed instructions about the disease, corticophobia and treatments by trained nurses, bathing and showering advices, adequate emollients use, avoidance of triggers (including possible and proven contact allergens), fingertip unit (FTU) explanation and a personalized scheme of topical therapy with topical corticosteroids (TCS) of different potencies, topical calcineurin inhibitors (TCI), and tar ointments, based on the recommendations on the use of topical therapy in national and international guidelines [6, 18, 20].

In the NB-UVB + OTT group, patients are allowed to apply topical therapies two hours after UV irradiation. Patients will use OTT for at least 3 months guided by standardized informational material. All patients will be instructed to keep empty tubes of their used topical treatment (excluding emollients) and to bring them to the appointments in order to analyze potency and quantities between the NB-UVB + OTT vs OTT group.

After 3 months of OTT, if needed, patients are allowed to take the next step in the AE treatment algorithm, being one of the currently available systemic immunomodulating therapies. During follow-up, patients from the OTT group are not allowed a NB-UVB course.

##### Rescue medication

For patients in the NB-UVB + OTT group, if sunburn occurs during NB-UVB, rescue medication consists of a one-time topical application of a class IV corticosteroid. For all patients, in case of severe uncontrolled AE during the study that requires rescue medication, a short course of oral prednisone can be prescribed [21]. Dosage and duration of this course will be determined by the treating physician. At the investigators’ discretion, rescue medication is allowed in other situations after consulting with the coordinating investigator.

##### Withdrawal of individual subjects

Patients can leave the study at any time for any reason if they wish to do so without consequences for their AE care. Investigators can decide to withdraw a patient from the study for urgent medical reasons. Reasons for withdrawal will be documented by the investigators. Withdrawal of participants has been accounted for in the sample size calculation.

If a participant prematurely discontinues study treatment, the patient will be encouraged to stay in the study to allow data collection at all remaining scheduled visits until completion of the planned end-of-study visit. The treating physician or research coordinator should aim to document the date of premature discontinuation from study treatment and the agreement of teh patient with collecting data and completing the remaining scheduled visits. Patients who fail the allocated therapy will, based on shared decision-making, continue with OTT or get screened and if eligible start with systemic immunomodulating therapy. During the follow-up period, patients will be monitored for effectiveness, safety, and satisfaction with the care received and have clinical assessments per schedule.

#### Outcomes

Outcomes and measurement instruments from the Core Outcome Set (COS) for AE clinical trials will be used [22]. The questionnaires can be found in Additional file 3.

##### Primary outcome

- Percentage of patients with EASI50 (a 50% decrease in EASI) at 3 months follow up (range EASI 0-72) [23, 24].

##### Secondary outcome

- Physician-reported clinical signs at 1–3–6–9–12 months:
  - Delta EASI
  - Percentage of patients with EASI50
  - vIGA-AD (range 0–4) [25].
  - Time until a 25% reduction in mean EASI is achieved
- Type, frequency, and quantity of topical therapies used
- Time to switch to systemic therapy
- Use, amount, and duration of rescue medication
- Patient-reported symptoms at 1–3–6–9–12 months:
  - PGA (Patient Global Assessment) (range 0–4) [26].
  - POEM (Patient-Oriented Eczema Measure) (range 0–28) [27, 28].
  - NRS-11 (Numeric Rating Scale) for peak itch over the past 24 h (range 0–10) [29].
- Patient-reported quality of life at 1–3–6–9–12 months:
  - DLQI (Dermatology Life Quality Index) (range 0–30) [30, 31].
- Patient-reported disease control at 1–3–6–9–12 months:
  - RECAP (recap of atopic eczema) (range 0–28) [32].
- Patient-reported satisfaction with received treatment:
  - PsoSat (Psoriasis care Satisfaction questionnaire) (range 0–30) [33].
- Cost-effectiveness at 3 and 12 months:
  - EQ-5D-5L (EuroQol 5D 5L) (range 11,111–55,555) [34].
  - Adapted iMCQ (iMTA Medical Consumption Questionnaire) [35].
  - Adapted iPCQ (iMTA Productivity Cost Questionnaire) [35].
- Percentage of patients who achieve Treatment Target goals for AE at 3 (EASI50, delta PGA ≥  − 1), 6–9–12 months (EASI ≤ 7.0, PGA ≤ 2) [36].
- Adverse events of special interest (AEoSIs) and serious adverse events (SAEs) at 1–3–6–9–12 months
- Percentage of therapy failures and dropouts and reasons at 1–3–6–9–12 months, categorized according to Medical Dictionary for Regulatory Activities (MedDRA).

#### Participant timeline

**Fig. 1:**
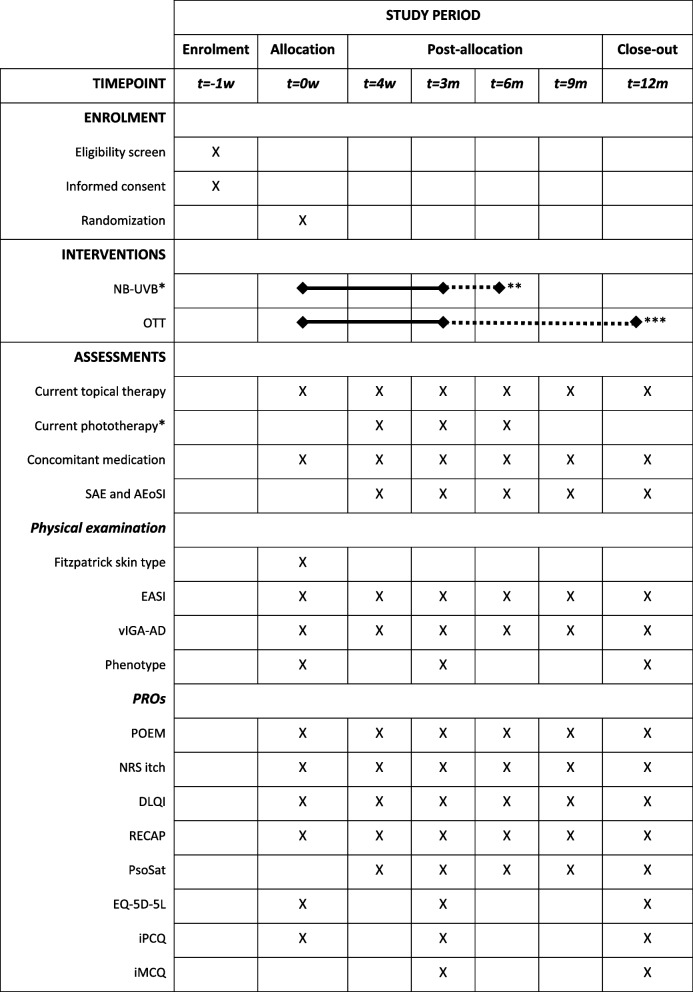
SPIRIT figure

After screening and informed consent, participants will be randomized in the NB-UVB + OTT or OTT group. Study participation will entail 6 study visits. The baseline visit will take place before therapy starts. The follow-up visits are at 4 weeks, 3, 6, 9, and 12 months. Visits may be rescheduled within 1 month of the initial planning. Extra visit(s) will take place in case of therapy switch or stop. Patients in the NB-UVB + OTT group will undergo phototherapy 3 times a week for a planned period of 12 weeks from baseline, with a possible range of 8 to 16 weeks, depending on therapy response. Therapy duration will be determined in accordance to standard clinical practice. NB-UVB can be applied in the outpatient clinic or at home. Participants in both groups will apply daily OTT for a minimum of 3 months. Patients will be asked to keep all tubes of used OTT (excluding emollients) and bring them to the study visits. During the visits, data about NB-UVB and OTT will be collected. Patients will be asked for possible SAEs and AEoSIs with details such as start and stop date, intensity/severity of adverse events, probability of relation with NB-UVB + OTT or OTT, whether the allocated therapy was interrupted, and co-medications. During all visits, a physical examination by a blinded outcome assessor will take place to assess disease severity. Additionally, a set of questionnaires will be sent to the patient. The questions can be filled out on an electronic device, either at home or during the study visit in the outpatient clinic. At baseline, 3 months, and 12 months, an additional set of questionnaires regarding disease burden on productivity and medical costs will be filled out (Fig. 1). Subjects that have completed trial participation or have discontinued prematurely will be referred back to standard eczema care.

#### Sample size

The required sample size is determined for the primary endpoint EASI50 at 3 months follow-up. Based on previous research published in the literature incidences of 50% effectiveness for reaching EASI50 using NB-UVB, and of 20–30% using only OTT are to be expected [37–40].

Sample sizes of 134 in both arms achieve 90% power to detect a difference between the group proportions of 20 percentage points. The proportion in the NB-UVB + OTT treatment group is assumed to be 30% under the null hypothesis and 50% under the alternative hypothesis. The assumed proportion in the control group treated with OTT alone is 30%. The test statistic used is the two-sided *Z*-test pooled variance. The significance level of the test is 5%. Interim analyses for early stopping will not be conducted; hence, no correction of the significance level for interim analyses is required. Allowing for a drop-out rate of up to 15%, a total of 316 patients will be recruited.

#### Recruitment

Subjects will be recruited from the patient population of each participating center. Additionally, recruitment will take place via the members of the Dutch Society of Dermatology and Venereology (NVDV) and the Dutch Association for People with Constitutional Eczema (VMCE). The investigators, NVDV members, and the VMCE board will be provided with promotional material that has been reviewed by the medical research ethics committee (MREC). In addition, MREC-evaluated promotional posters and texts will be distributed via NVDV- or VMCE-based media. This includes printed advertisements in magazines and digital promotion via sites/apps such as Facebook, Instagram, and LinkedIn.

### Randomization

Patients who meet eligibility criteria and who have provided written informed consent will be randomly allocated in a 1:1 ratio to either NB-UVB + OTT or OTT alone. To ensure allocation concealment, automated randomization is performed by the investigator using the electronic data management system Castor EDC. Randomization will use random permuted blocks.

### Blinding

During baseline- and follow-up visits, physician-reported outcomes (EASI, vIGA-AD) will be assessed. As these outcomes are subject to investigator bias, the physician outcome assessor will be blinded for treatment allocation. This independent (local) investigator will solely see the patient during the physical examination phase of the study visit. The patient will be instructed to not disclose their allocation outcome to this investigator. However, as NB-UVB may cause redness and tanning of the skin in light skin types, complete blinding of the outcome assessor will not be guaranteed. All other investigators or physicians performing study activities other than assessing EASI and vIGA-AD may be unblinded. The blinded outcome assessor may be unblinded when this is necessary to ensure patient safety.

### Data collection and management

All data is handled confidentially and in accordance with the European General Data Protection Regulation.

During baseline- and follow-up visits, physician-reported outcomes will be assessed by a blinded assessor who will note his/her findings on paper. This source document will be dated, signed, and kept on-site. All other study activities and the registration of physician-reported outcomes into the digital case report form (CRF) will be done by the investigator performing the study visit. Patient-reported outcomes will be entered by the participant into the electronic data management system via an invitation link, during or after every study visit. No biological specimens will be collected.

All patients will be assigned a unique subject identification number. Patients can be identified through a subject identification log and all data kept centrally will therefore be pseudonymized. This key linking patients with their identification number will be in possession of the investigators. Subject identification logs are kept on-site only and will not be shared centrally or with other sites.

Data will be collected in an electronic data management system: Castor Electronic Data Capture, from CIWIT BV, Amsterdam, the Netherlands: www.castoredc.com. Personnel responsible, e.g., data entry, monitoring, data validation, data exporting, and database administration, will have access to the database. Data handling will be described in the standard operating procedures (SOPs). All study documents will be stored securely in an environment only accessible to study team members. Data will be archived according to the local procedures of the sponsor. Data management was designed to adhere to the “FAIR Guiding Principles for scientific data management and stewardship,” aiming to optimize the Findability, Accessibility, Interoperability, and Reuse of digital assets [41].

### Statistical methods

#### Statistical methods for primary and secondary outcomes

The primary outcome measure will be estimated as a relative risk and absolute risk difference with 95% confidence interval and *p*-value. The primary outcome will be analyzed using a generalized linear model with binomial distribution and log-, or identity-link respectively. The primary analysis will be run using the intention-to-treat population. Missing data for the primary outcome is expected to be low, although meaningful drop-out can be expected.

A secondary analysis will be done using the per-protocol population. Exploratory subgroup analyses will be run, e.g., location of receiving NB-UVB (out-patient clinic or at home) and below or above the median EASI score at baseline.

Dichotomous secondary outcomes will be presented as counts, percentages, and relative risks according 95% confidence interval, and *p*-values will be calculated using the chi-square test or Fisher exact test as appropriate. Continuous variables will be presented as means with standard deviation when data distribution is normal. In case of skewed data medians with interquartile ranges will be used for presentation. The mean or median differences between the groups will be calculated with accompanying 95% confidence intervals using Satterthwaite’s or Hodges-Lehmann’s tests. Longitudinal data (repeated measures of, e.g., EASI, POEM, NRS-11, and EQ-5D-5L) will be analyzed using generalized estimating equations and adjusted for baseline values. Time to switching to systemic therapy will be analyzed as a time-to-event outcome using a Kaplan–Meier plot and log-rank test.

Multiple imputation will be used as necessary for secondary analyses and sensitivity analyses. The effects of intercurrent events will be assessed in sensitivity analyses. A detailed statistical analysis plan will be completed before the database lock.

The economic evaluation of NB-UVB as an add-on to OTT compared to OTT alone in this study population will be performed as a cost-utility analysis (CUA) from a societal perspective with the costs per quality-adjusted life year (QALY) as a primary outcome. Health utilities will be derived from the EQ-5D-5L health status profiles using the existing scoring algorithm [42]. The scoring algorithm is based on preferences in the general population for being in a particular health state, using the time trade-off elicitation technique. The health utility score may range from − 0.44 to 1.0, reflecting the worst, respectively, best health states possible. Deceased patients will be assigned a health utility score of 0. QALYs will be calculated as the area under the curve of health utility scores over time. In addition, a cost-effectiveness analysis (CEA) will be done with the costs per patient with a EASI50 as an outcome. The analysis will include the costs of health care (in-patient and out-patient hospital care, institutional care elsewhere, out-of-hospital care like general practitioner, and extramural drugs) and costs of productivity loss resulting from sick leave from work or lowered efficiency while at work. The volume of resources used will be gathered with clinical report forms and with patient questionnaires on medical consumption (iMCQ) and productivity loss (iPCQ) adapted for this study. No interim analyses are planned.

### Oversight and monitoring

#### Composition and responsibilities of the coordinating center and trial steering committee

This trial will be run by dermatologists, dermatological residents, (research) nurses, and PhD students in participating centers. They will be appointed, trained, and overseen by the trial steering committee consisting of the principal investigator, two project leads, a project coordinator, a methodologist, two experts in health technology assessment, a professional representative, a patient representative, and a representative of non-academic hospitals. The trial steering committee regularly meets with executive- and monitoring parties and produces progress reports to the sponsor and funders. The principal investigators, co-investigator, project coordinator, and methodologist conduct data management. All parties involved in the trial, including the participating centers, signed a consortium agreement that simultaneously serves as a clinical trial agreement. This legal document is kept in the trial master file and local investigator site files.

#### Monitoring

Monitoring will be performed in compliance with Good Clinical Practice (GCP), the guideline of the Dutch Federation of University Medical Centers (NFU), and additional regulations, if applicable. The trial sponsor appointed Trialbureau Zorgevaluatie Nederland (www.zorgevaluatienederland.nl/trialbureaus/trialbureau) as the party that will perform monitoring. An independent qualified monitor from this party will have access to the data and source documents of the study and will perform on-site or remote quality checks on the data collection, verification of data, the rights and wellbeing of the patients, and adherence to the study protocol. All sites will be visited by the monitor at least once during the study. Central remote monitoring will occur three months after the first included patient, followed by twice yearly. All monitoring arrangements and other details regarding quality assurance will be bundled in a separate monitoring plan.

#### Adverse event reporting

An adverse event is an undesirable experience occurring to a subject during the study, whether or not considered related to the study activities. As this study’s investigational therapy is already standard clinical practice, we theorize that it can be accurately predicted which adverse events are study related. The known short- and long-term side effects are considered mild. Therefore we consider it safe to not include all adverse events in this study’s dataset. Investigators will instead report the adverse events that are of special interest to this study. A full list can be found in Table 1. All adverse events will be categorized according to MedDRA.

**Table 1:** Adverse events of special interest

Adverse events of special interest (AEoSIs)
Sunburn
Photosensitive flare
Polymorfic light eruption
Burning and/or pruritus that requires management
Syncope
Development of skin cancer
Hyperpigmentation
Hypopigmentation
Contact allergy
Development of striae
Development of skin atrophy
Dermatitis perioralis/periorificialis
Folliculitis
Herpes simplex
Tinea incognito
Bacterial infection
Exacerbation of atopic eczema

##### Adverse events of special interest (AEoSI)

An adverse event of special interest is defined as an undesirable experience occurring to a subject during the study, whether or not considered related to the study activities, that is of interest to this study. All AEoSIs reported spontaneously by the subject or observed by the investigator or his staff will be recorded and analyzed (Table 1).

##### Serious adverse events (SAEs)

A serious adverse event is defined as any untoward medical occurrence or effect that.

- Results in death;
- Is life-threatening (at the time of the event);
- Requires hospitalization or prolongation of existing inpatients’ hospitalization;
- Results in persistent or significant disability or incapacity; and
- Any other important medical event that did not result in any of the outcomes listed above due to medical or surgical intervention but could have been based upon appropriate judgment by the investigator.

An elective hospital admission will not be considered as an SAE.

The investigator will report all SAEs to the sponsor (Amsterdam University Medical Centers) without undue delay after obtaining knowledge of the events. The sponsor will report the SAEs through the web portal ToetsingOnline to the accredited MREC that approved the protocol, within 7 days of first knowledge for SAEs that result in death or are life-threatening followed by a period of a maximum of 8 days to complete the initial preliminary report. All other SAEs will be reported within a period of a maximum of 15 days after the sponsor has first knowledge of the SAEs.

##### Follow-up of AEoSIs and SAEs

All AEoSIs and SAEs will be followed until they have abated, or until a stable situation has been reached. Depending on the event, follow-up may require additional tests or medical procedures as indicated, and/or referral to the general physician or a medical specialist. SAEs will be reported according to GCP guidelines until the study ends for the patient (when all 6 visits are completed, when the patient discontinues prematurely, or when the study is prematurely terminated).

#### Amendments

Amendments are changes made to the study after a favorable opinion by the accredited MREC has been given. The MREC that gave a favorable opinion will be notified of all amendments. Should amendments to the study protocol occur, an update will be given to the clinical trial registry. Occasional deviations from the study protocol will be fully documented using a breach report form.

#### Dissemination plans

The investigators will adhere to the statement on publication policy made by the Central Committee on Research Involving Human Subjects (CCMO). The results of this trial will be published in peer-reviewed international medical scientific journals, preferably open access, regardless of the conclusions drawn about the investigational therapy. For knowledge sharing, (preliminary) findings will be presented at (inter)national research meetings and conferences. Participants will be informed of the results through a lay summary. This summary will additionally be disseminated to patients through the Dutch patient association VMCE.

## Discussion

The UPDATE trial aims to provide high-quality evidence of the effectiveness, cost-effectiveness, and safety of NB-UVB in the short- and long-term when utilized as a treatment option for patients with moderate-to-severe AE. It will contribute to current knowledge of NB-UVB as a phototherapy modality for AE [14], and will be helpful to determine whether NB-UVB has a place in the current treatment algorithm for AE [6].

Assessing NB-UVB in the setting of a randomized trial poses several challenges. During the drafting of the study design, these challenges have been carefully identified and addressed.

The first challenge in the design of this study concerned the choice of the appropriate control group. Eligible study participants are AE patients who require a next step in care. Use of a no treatment or placebo control group would mean denying the control patients further management of their moderate-to-severe AE, and, therefore was considered unethical. Additionally, such a design will inevitably lead to a large number of drop-outs in the control group. Since the topical treatment of the study population still leaves room for improvement, we deem OTT with clear but pragmatic and personalized instructions, an appropriate choice of control group. Therefore, we consider a pragmatic trial in which all participants are treated with OTT and that investigates the benefit of NB-UVB as an add-on to OTT, the most appropriate choice [43]. With this design in a closer to real-life setting that enables all patients to benefit from trial participation, we believe that the UPDATE trial will provide high-quality data that is easily applicable to clinical practice.

Another challenge concerns the study patients and physicians being aware of allocated treatment. Blinding is an important component to ensure valid trial results and minimal bias. However, blinding of NB-UVB treatment is deemed logistically infeasible, as this would require a placebo (such as a blue light panel) that is not readily available in an outpatient- or at-home setting. Taking patient safety and -ethics into account, the current study design is considered the best possible option. By introducing an independent outcome assessor who is blinded to treatment allocation, physician-reported outcomes are in theory protected from confirmation bias. Additionally, patients will be instructed to not disclose their allocated treatment to the blinded investigator. In the assessment of NB-UVB, however, it may be visible to some degree to blinded researchers if a subject is or was receiving phototherapy. As NB-UVB may cause redness and tanning of the skin, complete blinding of the outcome assessor will not be guaranteed. This may affect lighter skin types more than darker skin types, as redness and tanning may be more visible.

In addition to the study limitations, the issue of reimbursement of NB-UVB should be considered. In the Netherlands, the process of retracting reimbursement is already in effect, leading to the out-phasing of NB-UVB as a treatment modality in the AE algorithm. This single trial may not suffice to provide compelling evidence to reverse this trend, but will need to be consolidated through pooling with other studies in an updated systematic review and possible meta-analysis. We expect with our UPDATE trial to importantly contribute to this matter.

## Trial status

Date of start recruitment

February 22nd 2023.

Date of completed recruitment (approximate)

July 1st 2024.

## Supplementary Information

Additional file 1: Dosing regimens for NB-UVB

Additional file 2: SPIRIT checklist

Additional file 3: Questionnaires

## Data Availability

The principal investigators, study coordinators, methodologists, and monitors of the project will have access to the final trial dataset. Direct access will be granted to authorized representatives from the sponsor, host institution, and the regulatory authorities to permit trial-related monitoring, audits, and inspections. All data required by this protocol will be made available upon request.

## Abbreviations

AE: Atopic eczema
AEoSI: Adverse event of special interest
BB: Broadband
BIA: Budget impact analysis
CCMO: Central Committee on Research Involving Human Subjects; in Dutch: Centrale Commissie Mensgebonden Onderzoek
COS: Core Outcome Set
CRF: Case report form
DALY: Disability-adjusted life years
DLQI: Dermatology Life Quality Index
DSMB: Data Safety Monitoring Board
EASI: Eczema Area and Severity Index
EQ-5D-5L: EuroQol 5D 5L
FTU: Fingertip unit
GCP: Good Clinical Practice
ICF: Informed consent form
IGA: Investigator Global Assessment
iMCQ: IMTA Medical Consumption Questionnaire
iPCQ: IMTA Productivity Cost Questionnaire
MedDRA: Medical Dictionary for Regulatory Activities
MREC: Medical research ethics committee
NB: Narrowband
NFU: Dutch Federation of University Medical Centers; in Dutch: Nederlandse Federatie van Universitair Medische Centra
NRS: Numeric Rating Scale
NVDV: Dutch Society of Dermatology and Venereology; in Dutch: Nederlandse Vereniging voor Dermatologie en Venerologie
OTT: Optimal topical therapy
PGA: Patient Global Assessment
POEM: Patient-Oriented Eczema Measure
PROBE: Prospective Randomized Open-label Blinded-Endpoint
PSOSAT: Psoriasis care Satisfaction questionnaire
PUVA: Photochemotherapy
RCT: Randomized controlled trial
RECAP: Recap of atopic eczema
(S)AE: (Serious) adverse event
SOP: Standard operating procedure
TCI: Topical calcineurin inhibitor
TCS: Topical corticosteroid
UK: United Kingdom
UVA1: Ultraviolet A-1
UVB: Ultraviolet B
VMCE: Association for People with Constitutional Eczema; in Dutch: Vereniging voor Mensen met Constitutioneel Eczeem
vIGA AD: Validated IGA for atopic dermatitis
