# Correction: The UPDATE trial (*UVB* *P*hototherapy in *D*ermatology for *At*opic *E*czema): study protocol for a randomized controlled trial of narrowband UVB with optimal topical therapy versus optimal topical therapy in patients with atopic eczema

**DOI:** 10.1186/s13063-025-08774-1

**Published:** 2025-02-17

**Authors:** Eva Knöps, Phyllis Spuls, Ruben Duijnhoven, Marcel Dijkgraaf, Marit van Barreveld, Bernd Arents, Annefoor van Enst, Floor Garritsen, Maruschka Merkus, Maritza Albertina Middelkamp-Hup, Annelie Musters, Angela Bosma, Ariënna Hyseni, Jitske Dijkstra, Dirk Jan Hijnen, Louise Gerbens

**Affiliations:** 1 Department of Dermatology, Amsterdam UMC, Location Academic Medi- Cal Center, Amsterdam Public Health, Infection and Immunity, University of Amsterdam, Amsterdam, The Netherlands; 2 Department of Obstetrics and Gynecology, Amsterdam UMC, Amsterdam Reproduction & Development Research Institute, University of Amsterdam, Amsterdam, the Netherlands; 3 Department of Epidemiology and Data Science, Amsterdam University Medi- Cal Centers, University of Amsterdam, Amsterdam, the Netherlands; 4 Dutch Association for People With Atopic Dermatitis, Nijkerk, the Netherlands; 5 Ned- Erlandse Vereniging Voor Dermatologie en Venereologie, NVDV, Utrecht, the Netherlands; 6 Department of Dermatology, HagaZiekenhuis, The Hague, the Netherlands; 7 Department of Dermatology, Erasmus MC University Medical Center, Rotterdam, the Netherlands

**Correction** **:** **Trials 25, 482 (2025)**

**https://doi.org/10.1186/s13063-024-08334-z**

Following the publication of the original article [1], we were notified that the 10^th^ author, originally published as Pina Middelkamp-Hup, should actually be Maritza Albertina Middelkamp-Hup.

The original article has now been corrected.
