# Supplementary material for: The UPDATE trial (UVB Phototherapy in Dermatology for ATopic Eczema): study protocol for a randomized controlled trial of narrowband UVB with optimal topical therapy versus optimal topical therapy in patients with atopic eczema
Source: Trials. 2024 Jul 16;25:482. doi: 10.1186/s13063-024-08334-z (PMC11253338; doi:10.1186/s13063-024-08334-z)
Supplement: Supplementary file 1 — Additional file 1: Dosing regimens for NB-UVB [file 13063_2024_8334_MOESM1_ESM.docx]

Fitzpatrick skin type I Fitzpatrick skin type II Fitzpatrick skin type III

Fitzpatrick skin type IV Fitzpatrick skin type V Fitzpatrick skin type VI

| **Step** | **J/cm^2^** |  |  |
| --- | --- | --- | --- |
| **1** | 0,05 | **23** | 0,74 |
| **2** | 0,10 | **24** | 0,81 |
| **3** | 0,11 | **25** | 0,90 |
| **4** | 0,12 | **26** | 0,98 |
| **5** | 0,13 | **27** | 1,08 |
| **6** | 0,15 | **28** | 1,19 |
| **7** | 0,16 | **29** | 1,31 |
| **8** | 0,18 | **30** | 1,44 |
| **9** | 0,19 |  |  |
| **10** | 0,21 |  |  |
| **11** | 0,24 |  |  |
| **12** | 0,26 |  |  |
| **13** | 0,29 |  |  |
| **14** | 0,31 |  |  |
| **15** | 0,35 |  |  |
| **16** | 0,38 |  |  |
| **17** | 0,42 |  |  |
| **18** | 0,46 |  |  |
| **19** | 0,51 |  |  |
| **20** | 0,56 |  |  |
| **21** | 0,61 |  |  |
| **22** | 0,67 |  |  |

| **Step** | **J/cm^2^** |  |  |
| --- | --- | --- | --- |
| **1** | 0,20 | **23** | 3,47 |
| **2** | 0,23 | **24** | 3,81 |
| **3** | 0,26 | **25** | 4,19 |
| **4** | 0,30 | **26** | 4,61 |
| **5** | 0,35 | **27** | 5,07 |
| **6** | 0,40 | **28** | 5,33 |
| **7** | 0,46 | **29** | 5,60 |
| **8** | 0,53 | **30** | 5,87 |
| **9** | 0,61 |  |  |
| **10** | 0,70 |  |  |
| **11** | 0,81 |  |  |
| **12** | 0,93 |  |  |
| **13** | 1,07 |  |  |
| **14** | 1,23 |  |  |
| **15** | 1,42 |  |  |
| **16** | 1,63 |  |  |
| **17** | 1,87 |  |  |
| **18** | 2,15 |  |  |
| **19** | 2,37 |  |  |
| **20** | 2,60 |  |  |
| **21** | 2,86 |  |  |
| **22** | 3,15 |  |  |

| **Step** | **J/cm^2^** |  |  |
| --- | --- | --- | --- |
| **1** | 0,25 | **23** | 3,96 |
| **2** | 0,29 | **24** | 4,36 |
| **3** | 0,33 | **25** | 4,80 |
| **4** | 0,38 | **26** | 5,28 |
| **5** | 0,44 | **27** | 5,54 |
| **6** | 0,50 | **28** | 5,82 |
| **7** | 0,58 | **29** | 6,11 |
| **8** | 0,67 | **30** | 6,41 |
| **9** | 0,76 |  |  |
| **10** | 0,88 |  |  |
| **11** | 1,01 |  |  |
| **12** | 1,16 |  |  |
| **13** | 1,34 |  |  |
| **14** | 1,54 |  |  |
| **15** | 1,77 |  |  |
| **16** | 2,03 |  |  |
| **17** | 2,24 |  |  |
| **18** | 2,46 |  |  |
| **19** | 2,71 |  |  |
| **20** | 2,98 |  |  |
| **21** | 3,28 |  |  |
| **22** | 3,60 |  |  |

| **Step** | **J/cm^2^** |  |  |
| --- | --- | --- | --- |
| **1** | 0,15 | **23** | 1,22 |
| **2** | 0,17 | **24** | 1,34 |
| **3** | 0,18 | **25** | 1,48 |
| **4** | 0,20 | **26** | 1,63 |
| **5** | 0,22 | **27** | 1,79 |
| **6** | 0,24 | **28** | 1,97 |
| **7** | 0,27 | **29** | 2,16 |
| **8** | 0,29 | **30** | 2,38 |
| **9** | 0,32 |  |  |
| **10** | 0,35 |  |  |
| **11** | 0,39 |  |  |
| **12** | 0,43 |  |  |
| **13** | 0,47 |  |  |
| **14** | 0,52 |  |  |
| **15** | 0,57 |  |  |
| **16** | 0,63 |  |  |
| **17** | 0,69 |  |  |
| **18** | 0,76 |  |  |
| **19** | 0,83 |  |  |
| **20** | 0,92 |  |  |
| **21** | 1,01 |  |  |
| **22** | 1,11 |  |  |

| **Step** | **J/cm^2^** |  |  |
| --- | --- | --- | --- |
| **1** | 0,30 | **23** | 5,79 |
| **2** | 0,36 | **24** | 6,08 |
| **3** | 0,43 | **25** | 6,39 |
| **4** | 0,52 | **26** | 6,71 |
| **5** | 0,62 | **27** | 7,04 |
| **6** | 0,75 | **28** | 7,40 |
| **7** | 0,90 | **29** | 7,77 |
| **8** | 1,07 | **30** | 8,15 |
| **9** | 1,29 |  |  |
| **10** | 1,55 |  |  |
| **11** | 1,86 |  |  |
| **12** | 2,23 |  |  |
| **13** | 2,45 |  |  |
| **14** | 2,70 |  |  |
| **15** | 2,97 |  |  |
| **16** | 3,26 |  |  |
| **17** | 3,59 |  |  |
| **18** | 3,95 |  |  |
| **19** | 4,34 |  |  |
| **20** | 4,78 |  |  |
| **21** | 5,26 |  |  |
| **22** | 5,52 |  |  |

| **Step** | **J/cm^2^** |  |  |
| --- | --- | --- | --- |
| **1** | 0,30 | **23** | 7,93 |
| **2** | 0,36 | **24** | 8,32 |
| **3** | 0,43 | **25** | 8,74 |
| **4** | 0,52 | **26** | 9,18 |
| **5** | 0,62 | **27** | 9,40 |
| **6** | 0,75 | **28** | 9,64 |
| **7** | 0,90 | **29** | 9,88 |
| **8** | 1,07 | **30** | 10,13 |
| **9** | 1,29 |  |  |
| **10** | 1,55 |  |  |
| **11** | 1,86 |  |  |
| **12** | 2,23 |  |  |
| **13** | 2,56 |  |  |
| **14** | 2,95 |  |  |
| **15** | 3,39 |  |  |
| **16** | 3,90 |  |  |
| **17** | 4,48 |  |  |
| **18** | 5,16 |  |  |
| **19** | 5,67 |  |  |
| **20** | 6,24 |  |  |
| **21** | 6,86 |  |  |
| **22** | 7,55 |  |  |
