# Supplementary material for: The UPDATE trial (UVB Phototherapy in Dermatology for ATopic Eczema): study protocol for a randomized controlled trial of narrowband UVB with optimal topical therapy versus optimal topical therapy in patients with atopic eczema
Source: Trials. 2024 Jul 16;25:482. doi: 10.1186/s13063-024-08334-z (PMC11253338; doi:10.1186/s13063-024-08334-z)
Supplement: Supplementary file 3 — Additional file 3: Questionnaires [file 13063_2024_8334_MOESM3_ESM.docx]

# Eczema Area and Severity Index - EASI

**Eczema Area and Severity Index (EASI) case report form - age≥8 years**

**Area of Involvement:** Each body region has potentially 100% involvement. Score **0 to 6** based on the following table:

| **% involvement** | **0** | **1-9%** | **10 - 29%** | **30 - 49%** | **50 - 69%** | **70 - 89%** | **90 - 100%** |
| --- | --- | --- | --- | --- | --- | --- | --- |
| **Region score** | **0** | **1** | **2** | **3** | **4** | **5** | **6** |

**Severity of Signs:** Grade the severity of each sign on a scale of **0 to 3**:

| **0** | **None**   - Take an average of the severity across the involved area. - Half points (1.5 and 2.5) may be used. 0.5 is not permitted – if a sign is present it should be at least mild (1) |
| --- | --- |
| **1** | **Mild** |
| **2** | **Moderate** |
| **3** | **Severe** |

**Scoring table:**

| **Body region** | **Erythema**  **(0-3)** | **Edema/**  **Papulation**  **(0-3)** | **Excoriation**  **(0-3)** | **Lichenification(0-3)** | **Region score**  **(0-6)** | **Multiplier** | **Score per body region** |  |
| --- | --- | --- | --- | --- | --- | --- | --- | --- |
| **Head/neck** | **( +** | **+** | **+** | **)** | **X** | **X 0.1** |  |  |
| **Trunk** | **( +** | **+** | **+** | **)** | **X** | **X 0.3** |  |  |
| **Upper extremities** | **( +** | **+** | **+** | **)** | **X** | **X 0.2** |  |  |
| **Lower extremities** | **( +** | **+** | **+** | **)** | **X** | **X 0.4** |  |  |
| ***The final EASI score is the sum of the 4 region scores:*** | | | | | | | **____________**  **(0-72)** | |


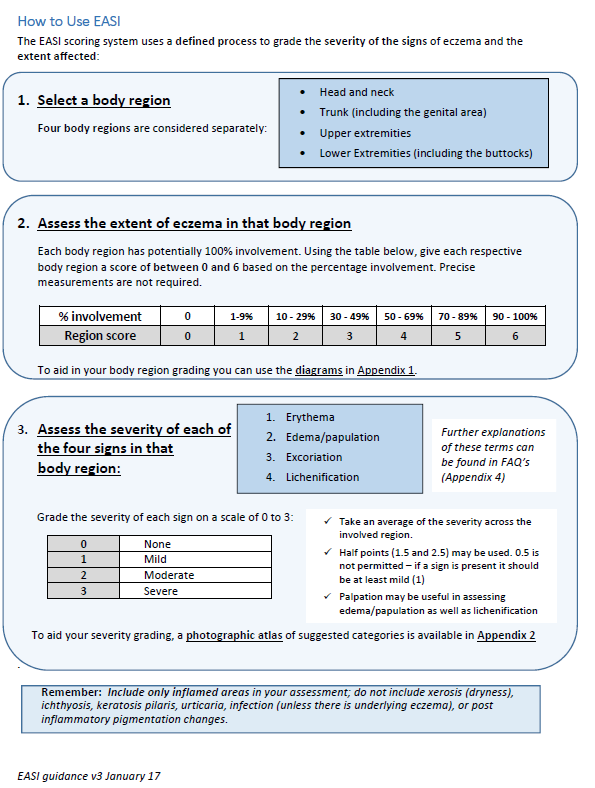


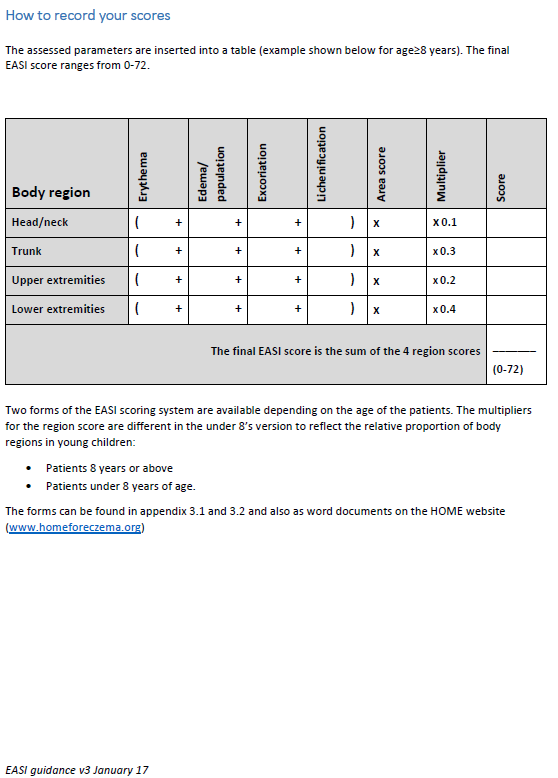


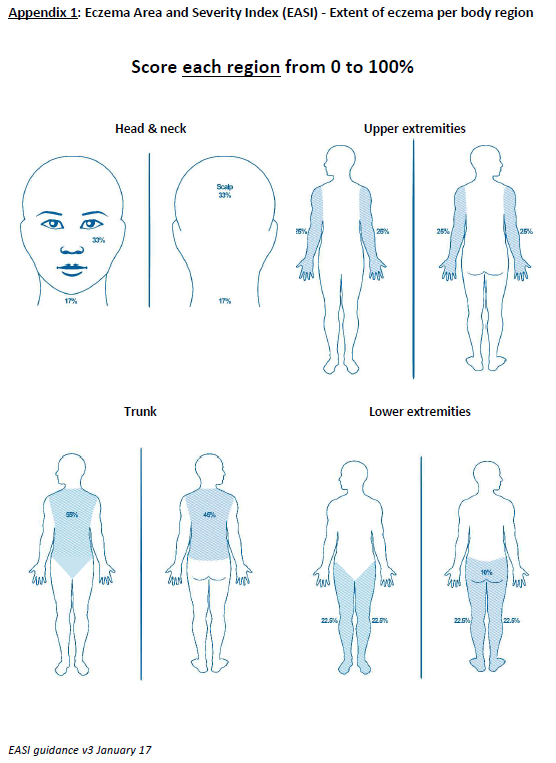


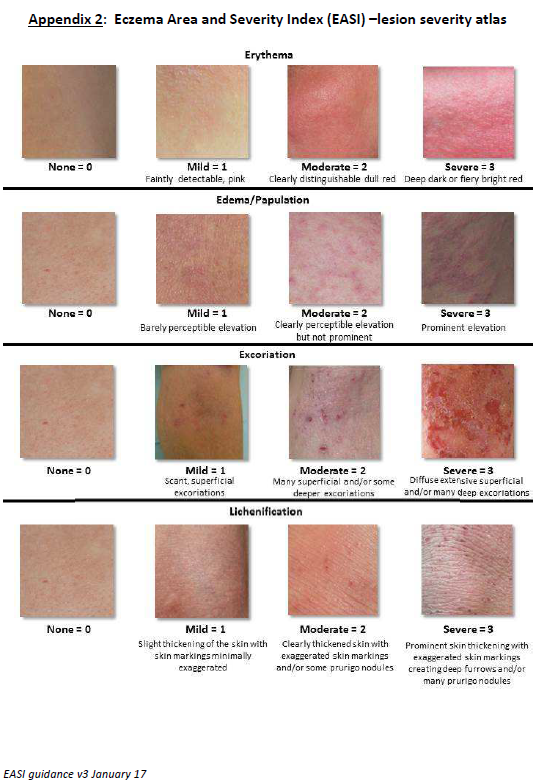


# Validated Investigator Global Assessment scale for Atopic Dermatitis - vIGA-AD


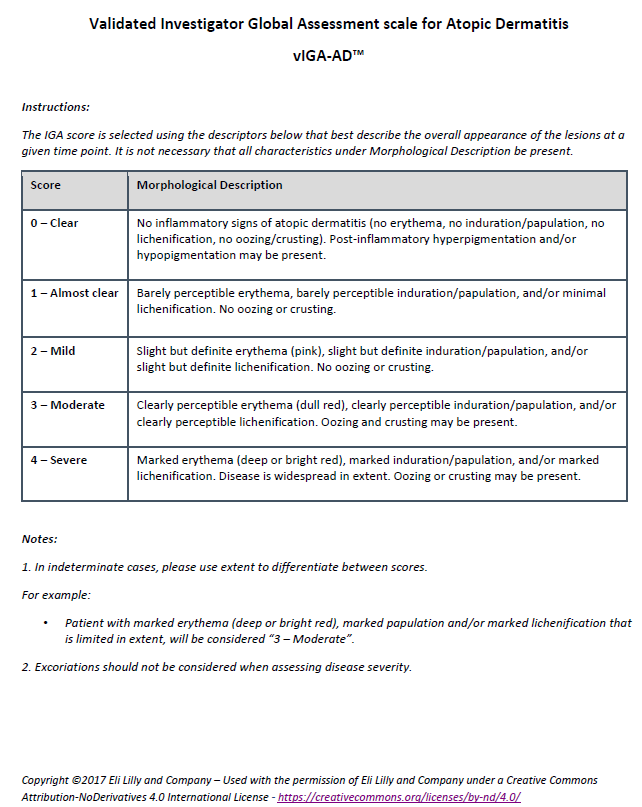


# Patient’s Global Assessment (English version) - PGA

Patient’s Global Assessment (English version)

(Own assessment of current eczema severity, circle what applies)

0 clear

1 almost clear

2 mild disease

3 moderate disease

4 severe disease

# Patient-Oriented Eczema Measure (English version) - POEM


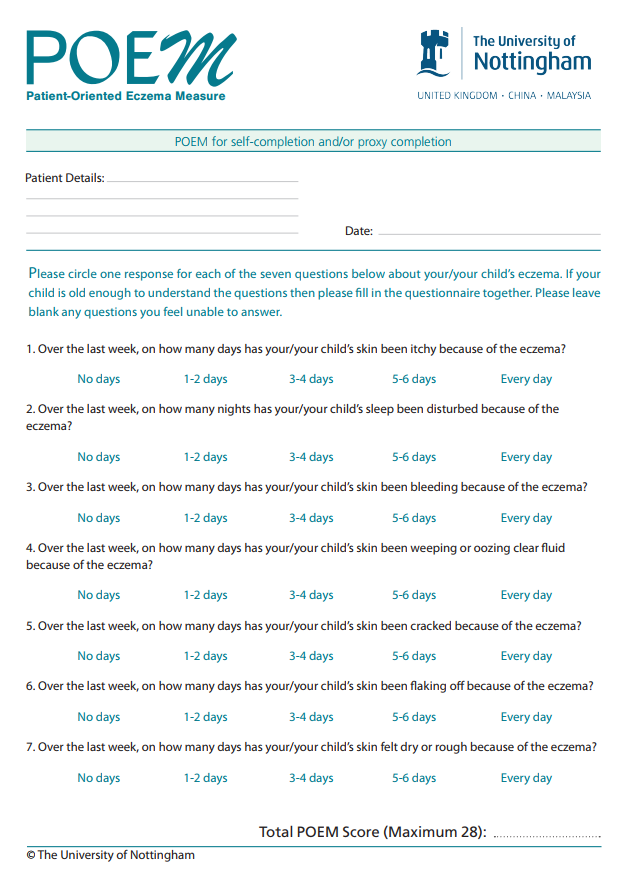


**
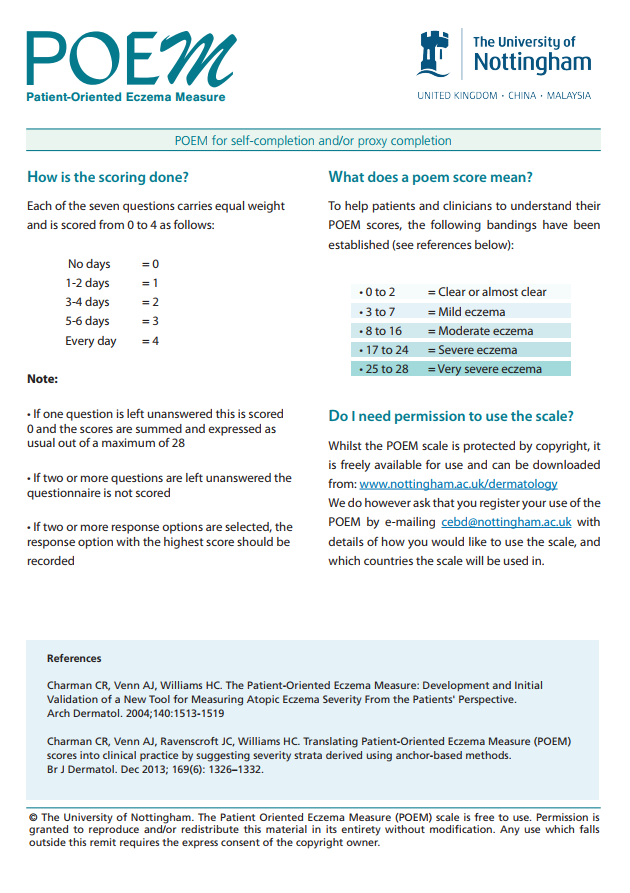
**

# Dermatology Life Quality Index (English version) - DLQI

**DERMATOLOGY LIFE QUALITY INDEX**

Date:

Patient ID: Score:

**The aim of this questionnaire is to measure how much your skin problem has affected your life OVER THE LAST WEEK. Please check one box for each question.**

| 1. | Over the last week, how **itchy**, **sore**, **painful** or **stinging** has your skin been? | Very much  A lot  A little  Not at all | 🞎  🞎  🞎  🞎 |  |
| --- | --- | --- | --- | --- |
| 2. | Over the last week, how **embarrassed** or **self conscious** have you been because of your skin? | Very much  A lot  A little  Not at all | 🞎  🞎  🞎  🞎 |  |
| 3. | Over the last week, how much has your skin interfered with you going **shopping** or looking after your **home** or **yard?** | Very much  A lot  A little  Not at all | 🞎  🞎  🞎  🞎 | Not relevant 🞎 |
| 4. | Over the last week, how much has your skin influenced the **clothes** you wear? | Very much  A lot  A little  Not at all | 🞎  🞎  🞎  🞎 | Not relevant 🞎 |
| 5. | Over the last week, how much has your skin affected any **social** or **leisure** activities? | Very much  A lot  A little  Not at all | 🞎  🞎  🞎  🞎 | Not relevant 🞎 |
| 6. | Over the last week, how much has your skin made it difficult for you to do any **sport**? | Very much  A lot  A little  Not at all | 🞎  🞎  🞎  🞎 | Not relevant 🞎 |
| 7. | Over the last week, has your skin prevented you from **working** or **studying**? | yes  no | 🞎  🞎 | Not relevant 🞎 |
|  | If "No", over the last week how much has your skin been a problem at **work** or **studying**? | A lot  A little  Not at all | 🞎  🞎  🞎 |  |
| 8. | Over the last week, how much has your skin created problems with your **partner** or any of your **close friends** or **relatives**? | Very much  A lot  A little  Not at all | 🞎  🞎  🞎  🞎 | Not relevant 🞎 |
| 9. | Over the last week, how much has your skin caused any **sexual difficulties**? | Very much  A lot  A little  Not at all | 🞎  🞎  🞎  🞎 | Not relevant 🞎 |
| 10. | Over the last week, how much of a problem has the **treatment** for your skin been, for example by making your home messy, or by taking up time? | Very much  A lot  A little  Not at all | 🞎  🞎  🞎  🞎 | Not relevant 🞎 |

**Please check you have answered EVERY question. Thank you.**

©AY Finlay, GK Khan, April 1992, This must not be copied without the permission of the authors.

# NRS PEAK PRURITUS PAST 24 HOURS (Dutch version) - PEAK NRS

**On a scale of 0 to 10, with 0 being ‘no itch’ and 10 being the ‘worst itch imaginable’, how would you rate your itch at the worst moment during the previous 24 hours?**


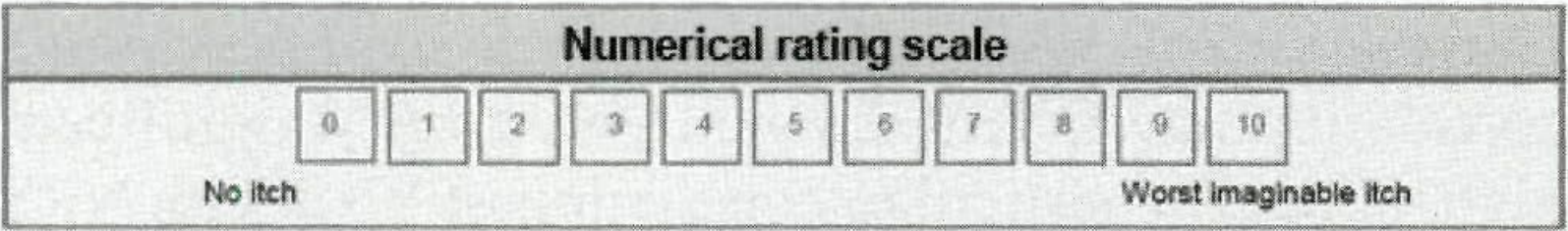


NRS PEAK PRURITUS PAST 24 HOURS (English version)

**Op een schaal van 0 tot 10, waarbij 0 ‘geen jeuk’ en 10 ‘ergst denkbare jeuk’ betekent, welk cijfer zou u geven aan de ergste jeuk (de piek) van afgelopen 24 uur?**


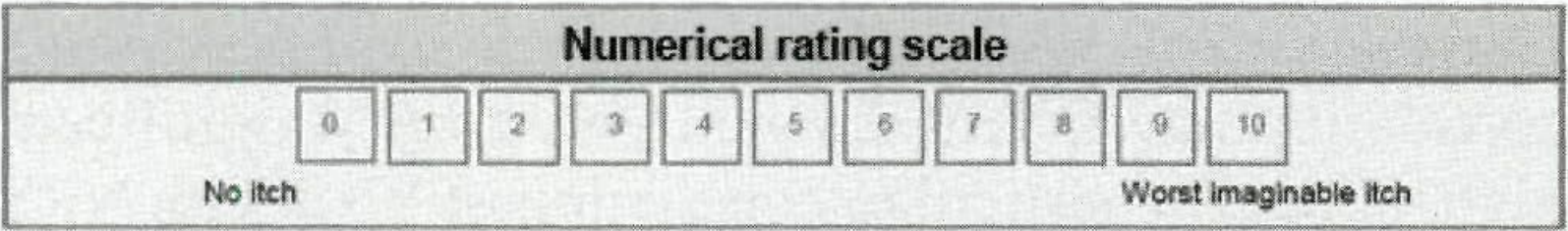


Reference:

Phan NQ, Blome C, Fritz F, Gerss J, Reich A, Ebata T, Augustin M, Szepietowski JC, Ständer S. **Assessment of pruritus intensity: prospective study on validity and reliability of the visual analogue scale, numerical rating scale and verbal rating scale in 471 patients with chronic pruritus.** *Acta Derm Venereol.* 2012 Sep;**92**(5):502-7.

# Recap of atopic eczema (English version) - RECAP
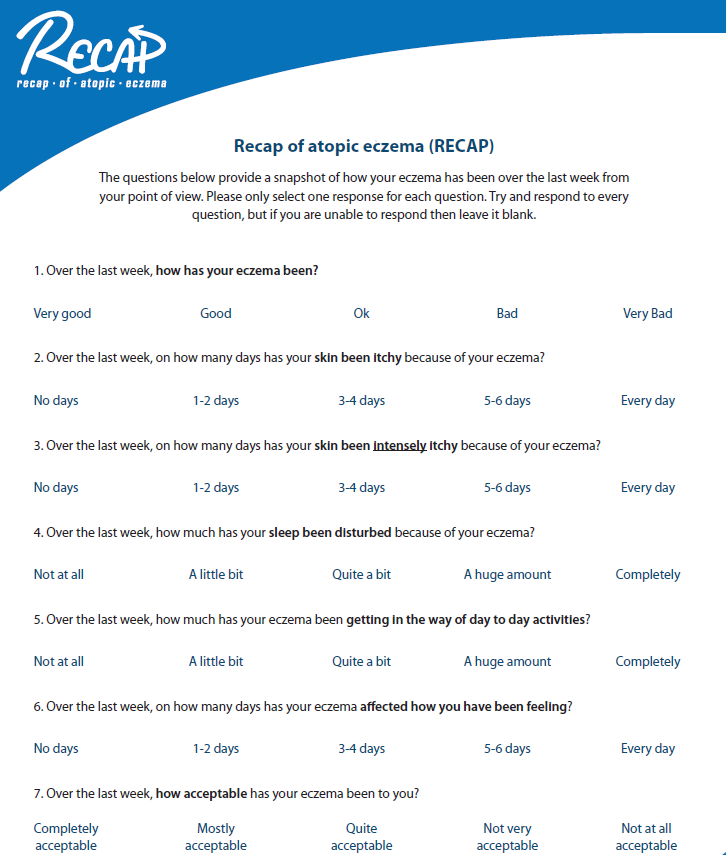


#
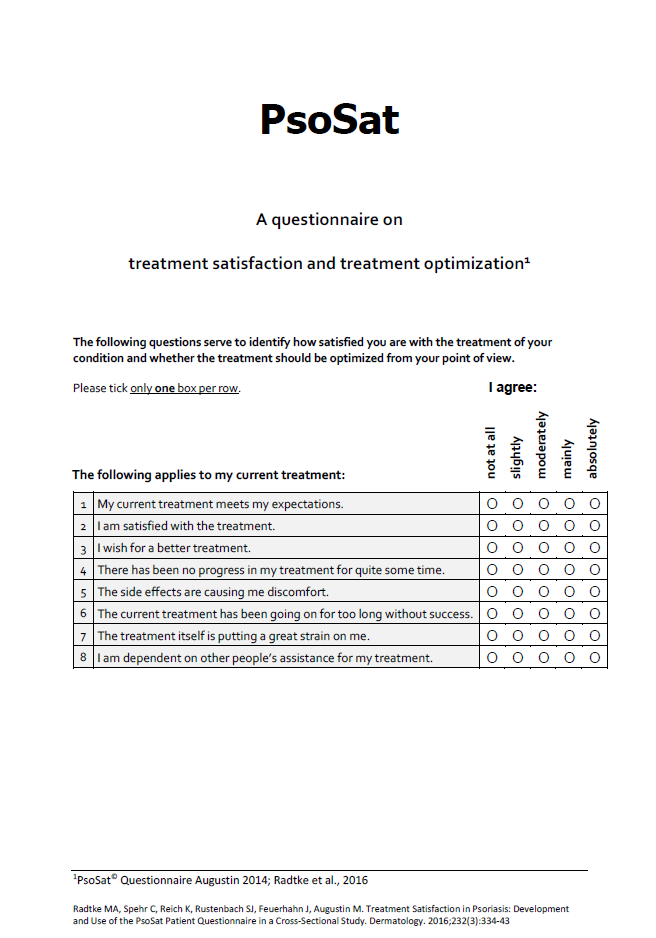
Psoriasis care satisfaction questionnaire (English version only) - PsoSat

# EuroQol-5 dimensions-5 level (English version) – EQ-5D-5L


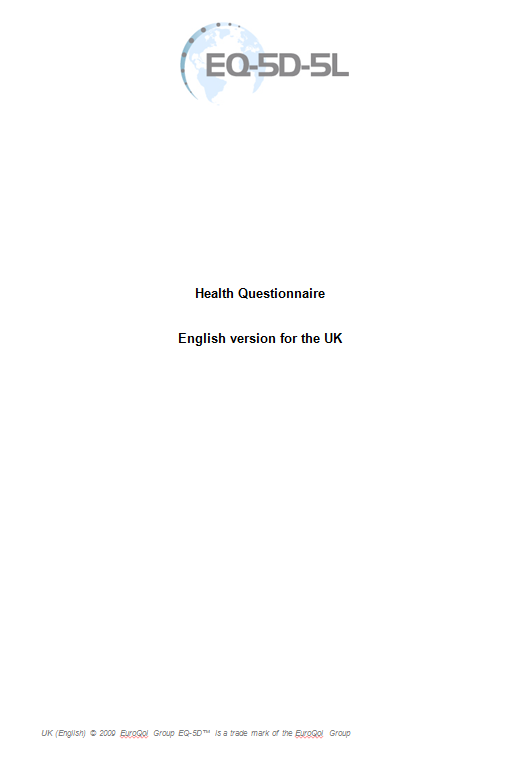


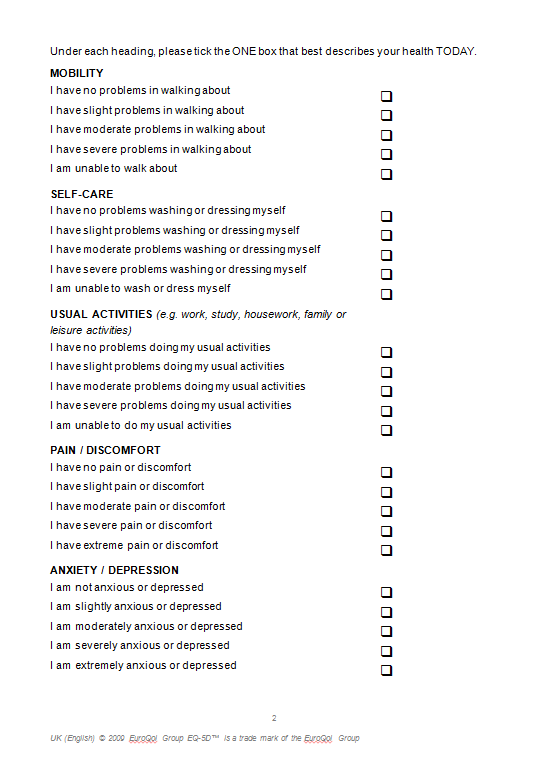


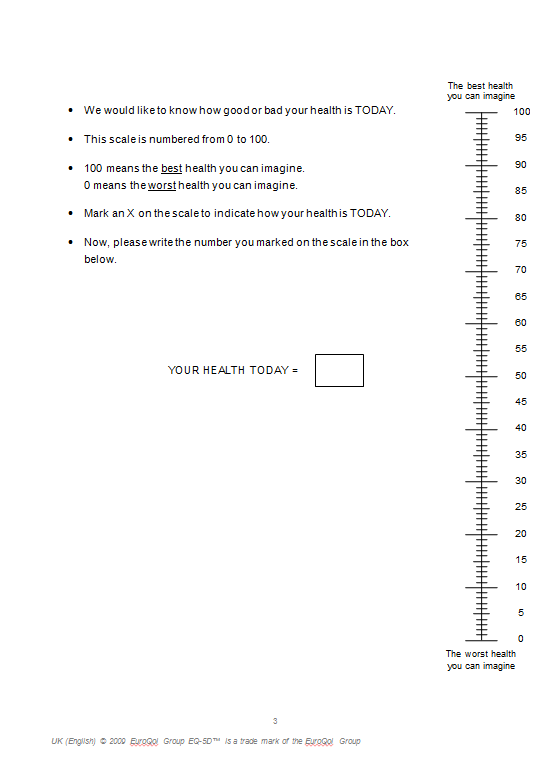


iMTA Productivity Cost Questionnaire (English version) – iPCQ baseline

**Please read this first!**

**What is the questionnaire about?**

The questionnaire is about your health and work in the past 4 weeks. We will start with general questions.

**How long does it take to complete the questionnaire?**

It takes roughly 5 minutes to complete the questionnaire.

**How should you complete the questionnaire?**

- Start with the first question and follow the numbering.

- Check 1 box for each question, unless the question says that you can check more than 1 box.

- For some questions, you can enter a number or something else.

- There are no wrong answers.

**Question A1. On what date are you completing this questionnaire?**

.... …. …. …. …. …. …. ….

Date Month Year

**Question A2. What is your occupation?** Check the box for what best describes your primary occupation.

□ I am at school, I study

□ I am in paid employment

□ I am self-employed

□ I am a housewife/househusband

□ I am unemployed

□ I am disabled for working, for, … % (bodily percentage)

□ I am retired or have taken early retirement

□ I do something else, namely …………………………………..

**Question A3. Do you have paid work?**

□ No 🡪 Thank you for completing this questionnaire.

□ Yes 🡪 Go to question 1, 2 and 3.

**Question 1. What is your occupation?**

..………………………………………………………………….

**Question 2. How many hours a week do you work?** Add together all the hours for which you are paid.

…… hours

**Question 3. How many days a week do you work?**

…… days

Thank you for completing this questionnaire.

iMTA Productivity Cost Questionnaire (English version) – iPCQ follow-up

**Please read this first!**

**What is the questionnaire about?**

The questionnaire is about your health and work in the past 4 weeks. We will start with general questions.

**How long does it take to complete the questionnaire?**

It takes roughly 10 minutes to complete the questionnaire.

**How should you complete the questionnaire?**

- Start with the first question and follow the numbering.

- Check 1 box for each question, unless the question says that you can check more than 1 box.

- For some questions, you can enter a number or something else.

- There are no wrong answers.

**Question A1. On what date are you completing this questionnaire?**

.... …. …. …. …. …. …. ….

Date Month Year

**Question A2. What is your occupation?** Check the box for what best describes your primary occupation.

□ I am at school, I study

□ I am in paid employment

□ I am self-employed

□ I am a housewife/househusband

□ I am unemployed

□ I am disabled for working, for, … % (bodily percentage)

□ I am retired or have taken early retirement

□ I do something else, namely …………………………………..

**Question A3. Do you have paid work?**

□ No 🡪 Thank you for completing this questionnaire.

□ Yes 🡪 Go to question 1, 2 and 3.

**Question 1. What is your occupation?**

..………………………………………………………………….

**Question 2. How many hours a week do you work?** Add together all the hours for which you are paid.

…… hours

**Question 3. How many days a week do you work?**

…… days

**Question 4. Have you been absent from your work in the past 4 weeks because you were ill?**

□ No 🡪 Go to question 7.

□ Yes, I was absent for….. days 🡪 Go to question 5.

*(Only count the working days in the past 4 weeks)*

**Question 5. Have you been absent from your work because of being ill for longer than the entire period of 4 weeks?** This refers to an uninterrupted period of absence from work.

□ No 🡪 Go to question 7.

□ Yes 🡪 Go to question 6.

**Question 6. When did you report being ill?**

.... …. …. …. …. …. …. ….

Day Month Year 🡪 Thank you for completing this questionnaire.

**Question 7. Have there been days over the past 4 weeks when you worked but suffered from physical or psychological problems during your work?**

□ No 🡪 Thank you for completing this questionnaire.

□ Yes 🡪 Go to question 8.

**Question 8. On how many working days have you suffered from physical or psychological problems during your work?** Just count the working days over the past 4 weeks.

…… working days

**Question 9. On the days when you were suffering from problems, perhaps you were not able to do as much work as normal. On those days, how much work could you do on average?** Look at the numbers below. A 10 means that you were able to do just as much as normal on those days. A 0 means that you were not able to do anything on those days. Circle the right number.

| I was not able to do anything on those days | |  |  |  | I could do around half | |  |  |  | I was able to do just as much as normal | |
| --- | --- | --- | --- | --- | --- | --- | --- | --- | --- | --- | --- |
| 0 | 1 | 2 | 3 | 4 | 5 | 6 | 7 | 8 | 9 | 10 |  |

Thank you for completing this questionnaire.

iMTA Medical Consumption Questionnaire (English version) - iMCQ

**Please read this first!**

**What is the questionnaire about?**

The questionnaire is about your use of care in the past 3 months.

**How long does it take to complete the questionnaire?**

It takes roughly 20 minutes to complete the questionnaire.

**How should you complete the questionnaire?**

- Start with the first question and follow the numbering.

- Check 1 box for each question, unless the question says that you can check more than 1 box.

- For some questions, you can enter a number or something else.

- There are no wrong answers.

**Question 1. On what date are you completing this questionnaire?**

.... …. …. …. …. …. …. ….

Date Month Year

**Question 2. How many appointments did you have with your GP (general practitioner) in the past 3 months?**

□ No appointment

□ …… appointments

**Question 3. How many appointments did you have with your nurse practitioner in the past 3 months?**

□ No appointment

□ …… appointments

**Question 4. How many appointments did you have with a psychologist in the past 3 months?**

□ No appointment

□ …… appointments

**Question 5. How many appointments did you have with the company doctor in the past 3 months?**

□ No appointment

□ …… appointments

**Question 6. Have you received home care in the past 3 months?**

□ No 🡪 Go to question 8.

□ Yes 🡪 Go to question 7.

**Question 7. What kind home care have you had in the past 3 months? How many hours of home care did you receive on average?**

You can tick more than 1 box.

Housekeeping and domestic help *example: vacuuming, making bed, going for daily groceries*

□ No

□ Yes …. hours a week

Personal care *example: help with bathing or dressing*

□ No

□ Yes …. hours a week

Nursing *example: putting on a bandage, administering medication, measuring blood pressure*

□ No

□ Yes …. hours a week

**Question 8. How many times did you visit the emergency room of a hospital for the past 3 months?**

□ Not once

□ …… times

**Question 9. Did you have an appointment at the outpatient clinic of the hospital in the past 3 months with one of the following doctors? And how many?**

Dermatologist (outside the study you are participating in) □ No □ Yes, … times

Ear-nose-throat-doctor (ENT-doctor) □ No □ Yes, … times

Pulmonologist (lung doctor) □ No □ Yes, … times

Ophthalmologist (eye doctor) □ No □ Yes, … times

Allergist (allergy doctor) □ No □ Yes, … times

**Question 10. Did you visit the hospital for day care treatment during the past 3 months?** So you did not stay overnight. Examples of day care treatments are blood transfusions, renal dialysis or a chemo course.

□ No 🡪 Go to question 12.

□ Yes 🡪 Go to question 11.

**Question 11. For what kind of treatment was this?** Was this for more than one type of treatment? Then enter all types of treatments.

Treatment 1: ..………………………………… I had to go here … times in the last 3 months

Treatment 2: ..………………………………… I had to go here … times in the last 3 months

Treatment 3: ..………………………………… I had to go here … times in the last 3 months

**Question 12. Have you been admitted to a hospital for inpatient care in the past 3 months?** So you had to stay overnight. For example, because you had surgery and could not go home immediately.

□ No 🡪 Go to question 15.

□ Yes 🡪 Go to question 13.

**Question 13. How often have you been admitted to the hospital for inpatient care in the past 3 months?**

...... times in the past 3 months

**Question 14. How long have you stayed in the hospital?** Have you been in the hospital more than once in the past 3 months? Then add all the days together.

...... days in total in the past 3 months, of which …. days in critical care (also called intensive care)

**Question 15. Did you have other medical expenses in the past 3 months that you did not receive a reimbursement for?**

Over the counter drugs

□ No

□ Yes approximately …. euros per month

Specialty foods

□ No

□ Yes approximately …. euros per month

Home care

□ No

□ Yes approximately …. euros per month
